# *Helicobacter pylori* infection increase the risk of subclinical hyperthyroidism in middle-aged and elderly women independent of dietary factors: Results from the Tianjin chronic low-grade systemic inflammation and health cohort study in China

**DOI:** 10.3389/fnut.2023.1002359

**Published:** 2023-03-06

**Authors:** Juanjuan Zhang, Xinghua Hai, Siyu Wang, Fan Zhu, Yeqing Gu, Ge Meng, Qing Zhang, Li Liu, Hongmei Wu, Shunming Zhang, Tingjing Zhang, Xing Wang, Shaomei Sun, Ming Zhou, Qiyu Jia, Kun Song, Kaijun Niu

**Affiliations:** ^1^Nutritional Epidemiology Institute and School of Public Health, Tianjin Medical University, Tianjin, China; ^2^First Teaching Hospital of Tianjin University of Traditional Chinese Medicine, Tianjin, China; ^3^School of Public Health of Tianjin University of Traditional Chinese Medicine, Tianjin, China; ^4^Institute of Radiation Medicine, Chinese Academy of Medical Sciences & Peking Union Medical College, Tianjin, China; ^5^Health Management Centre, Tianjin Medical University General Hospital, Tianjin, China; ^6^Tianjin Key Laboratory of Environment, Nutrition and Public Health, Tianjin, China; ^7^Center for International Collaborative Research on Environment, Nutrition and Public Health, Tianjin, China

**Keywords:** *Helicobacter pylori*, subclinical hyperthyroidism, dietary factors, propensity score matching, cohort study

## Abstract

**Background:**

Prospective studies on the association between *Helicobacter pylori* (*H. pylori*) infection and subclinical hyperthyroidism are limited. We, therefore, designed a large-scale cohort study to explore the association between *H. pylori* infection and the risk of subclinical hyperthyroidism in women.

**Methods:**

This prospective cohort study investigated 2,713 participants. *H. pylori* infection was diagnosed with the carbon 13 breath test. Subclinical hyperthyroidism was defined as serum thyroid-stimulating hormone levels are low or undetectable but free thyroxine and tri-iodothyronine concentrations are normal. Propensity score matching (PSM) analyses and Cox proportional hazards regression models were used to estimate the association between *H. pylori* infection and subclinical hyperthyroidism.

**Results:**

A total of 1,025 PS-matched pairs of *H. pylori* infection women were generated after PSM. During 6 years of follow-up, the incidence rate of subclinical hyperthyroidism was 7.35/1,000 person-years. After adjusting potential confounding factors (including iodine intake in food and three main dietary patterns score), the multivariable hazard ratio (HR; 95% confidence intervals) of subclinical hyperthyroidism by H. pylori infection was 2.49 (1.36, 4.56). Stratified analyses suggested a potential effect modification by age, the multivariable HR (95% confidence intervals) was 2.85 (1.45, 5.61) in participants aged ≥ 40 years and 0.70 (0.08, 6.00) in participants aged < 40 years (P for interaction = 0.048).

**Conclusion:**

Our prospective study first indicates that *H. pylori* infection is significantly associated with the risk of subclinical hyperthyroidism independent of dietary factors among Chinese women, especially in middle-aged and older individuals.

**Clinical Trial Registration:**https://upload.umin.ac.jp/cgi-open-bin/ctr_e/ctr_view.cgi?recptno=R000031137, identifier UMIN000027174.

## Introduction

Thyroid hormones (THs), including triiodothyronine (T3) and thyroxine (T4), are produced and released by the thyroid gland (1). They are key regulators of basal metabolic rate and are essential for the normal growth and development of a body. Synthesis and secretion of THs are finely modulated by the hypothalamic–pituitary–thyroid (HPT) axis. Thyroid-stimulating hormone (TSH) is produced by the anterior pituitary, stimulating the synthesis and secretion of THs by negative feedback inhibition of the HPT axis (2). Subclinical hyperthyroidism (SHyper) is defined biochemically: TSH concentrations are low or undetectable but free thyroxine (FT4) and tri-iodothyronine (FT3) concentrations are normal (3). The common causes of subclinical hyperthyroidism include toxic multinodular goiter, toxic adenoma, Graves’ disease, and some exogenous causes (4). The prevalence of subclinical hyperthyroidism in the general population is 1%–3% (5, 6), which varies by age, sex, race, genetic predisposition, iodine status, and the definition of subclinical hyperthyroidism (7). Women, older individuals, and those living in iodine-deficient regions were more frequent (8). In the past decade, the adverse effects of subclinical hyperthyroidism on health have been explored extensively, which has previously been associated with an increased risk for cardiovascular disease, bone loss, fractures, and dementia, even may progression to overt hyperthyroidism (4), which have become an important public health problem.

*Helicobacter pylori* (*H. pylori*) is a major human pathogen that specifically colonizes the gastric epithelium and infects the stomach of 44.3% (50.8% in developing countries vs. 34.7% in developed countries) of the world population (9, 10). Although most infected individuals do not develop obvious clinical sequelae, *H. pylori* is a Group I carcinogen according to the International Agency for Research on Cancer (IARC), with 89% of all gastric cancers being attributable to this infection (9). *Helicobacter pylori* infection can also involve some extra gastric diseases, such as respiratory (bronchiectasis and asthma), cardiovascular (atherosclerosis, myocardial infarction), Parkinson’s disease, metabolic syndrome, fatty liver disease, and immune-mediated allergic diseases (11, 12). Evidence suggests that *H. pylori* infection plays an important role in the pathogenesis of thyroid autoimmune diseases due to its ability to mimic antigen distribution on thyroid cell membranes (13, 14). In addition, De Luis et al. (15) showed that the titer of anti- *H. pylori* immunoglobulin G (IgG) antibody in patients with subclinical hyperthyroidism was significantly higher than that in the control group. Furthermore, a recent meta-analysis (16) involving 862 patients showed that *H. pylori* infection was significant in Graves’ disease but not in Hashimoto thyroiditis.

To date, specific data linking *H. pylori* infection with subclinical hyperthyroidism in human populations has been limited and conflicting, and most of the thyroid diseases show woman predilection (17), therefore, we conducted a large prospective cohort study to investigate whether baseline *H. pylori* infections were associated with subclinical hyperthyroidism in woman adults. Moreover, evidence has illustrated that dietary factors may play a crucial role in subclinical hyperthyroidism (18), which also directly influences *H. pylori* colonization or virulence (19). Therefore, while studying the association between *H. pylori* infection and subclinical hyperthyroidism, we must consider the interference of dietary factors in the association. Owing to it being a retrospective cohort, propensity score matching (PSM) was applied to reduce selection bias when comparing the clinical outcomes between patients with and without *H. pylori* infections.

## Materials and methods

### Participants

Tianjin Chronic Low-grade Systemic Inflammation and Health (TCLSIH) Cohort Study is a prospective dynamic cohort study of participants over 18 years of age, focusing on the relationship between chronic low-grade systemic inflammation and health status (20, 21). This is an ongoing study that was launched in 2007, where the participants underwent health examinations and completed a questionnaire survey to assess their diet and lifestyle factors till May 2013 (22). Refer to previous reports for detailed information (23).

**Figure 1:**
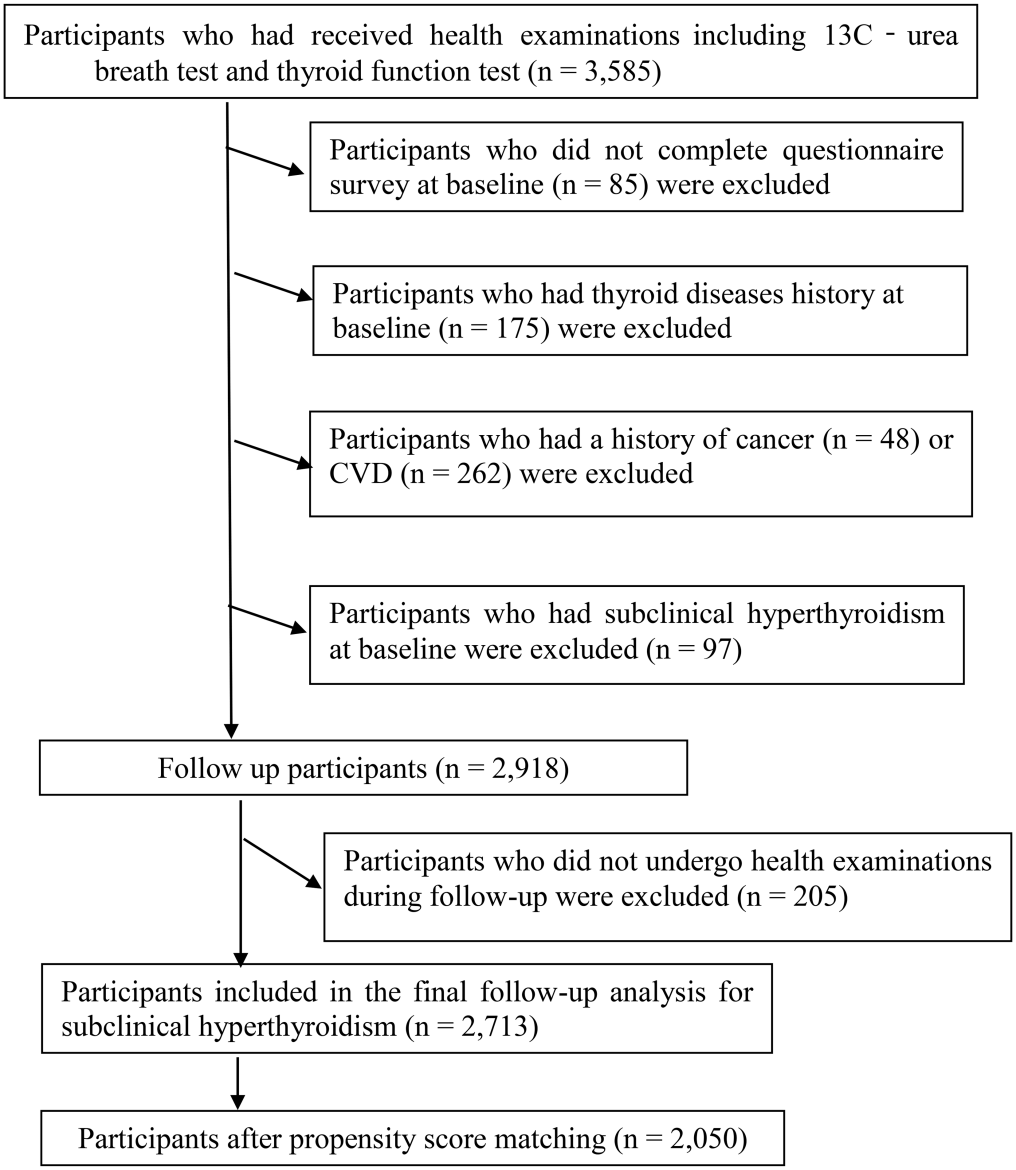
Flow diagram of study participant selection.

A total of 3,585 women had received at least one health examination and questionnaire survey, including blood tests, thyroid function tests, 13C-urea breath test (^13^C-UBT), and lifestyle factors. During the research period. Participants who did not complete the questionnaire survey at baseline (*n* = 85) were excluded. In addition, we excluded participants who had a history of cardiovascular disease (*n* = 262) or cancer (*n* = 48), given that cardiovascular disease and cancer can significantly affect the lifestyle of participants. We also excluded participants who had a history of thyroid disease (*n* = 175) at baseline, subjects with subclinical hyperthyroidism at baseline (*n* = 97), and 205 participants who were lost to follow-up examinations. The final cohort analysis comprised 2,713 participants (follow-up rate, 93.0%). The participant selection process is described in Figure 1. All participants have agreed to participate and provided written informed consent. The protocol of the study is in accordance with the Declaration of Helsinki and was approved by the Institutional Review Board of Tianjin Medical University.

### Thyroid function tests

After fasting for 8–12 h, fasting blood samples were collected by elbow vein puncture in the morning. Serum FT3, FT4, and TSH were measured by chemiluminescence immunoassay using the ADVIA Centaur FT3, FT4, and TSH3-Ultra analyzer (Siemens Healthcare Diagnostics, New York, NY). The ranges of measurement for FT3, FT4, and TSH were 0.3–30.8 and 1.3–155 pmol/L, and 0.001–150 mIU/L, respectively. Based on the previous reports and national guidelines (23, 24), the normal ranges of FT3, FT4, and TSH were 3.50–6.50 and 11.50–22.70 pmol/L, and 0.55–4.78 mIU/L, respectively. We used a uniform thyrotropin cutoff level for subclinical hyperthyroidism definition: TSH < 0.55 IU/mL and normal FT4 levels.

### Assessment of *Helicobacter pylori* infection

The diagnosis of *H. pylori* infection was based on the result of fasting ^13^C-UBT, which is based on the simple principle that the abundantly expressed urease of *H. pylori* can rapidly hydrolyze isotopically labeled urea solutions. A baseline breath sample was obtained by blowing into a 20-mL container, and a capsule containing 75 mg of ^13^C-urea was given to patients with 100 ml of water. Two breath samples were collected within a 30-min interval. Patient samples were analyzed by gas chromatography and *H. pylori* infection was considered present if the difference between the 30-min value and baseline value divided by the baseline value exceeded 4.0‰. ^13^C-UBT is a mature diagnostic test for *H. pylori* infection due to its rapid, non-invasive, high sensitivity, and specificity (25).

### Assessment of other variables

Data on potential confounders-such as demographic characteristics, socioeconomic status, lifestyle factors, reproductive factors, and family history of CVD, hypertension, diabetes, and hyperlipidemia were obtained from a standardized health-related questionnaire survey. The total energy intake for each participant and dietary intake was assessed using a 100-item Food Frequency Questionnaire (FFQ). Spearman rank correlation coefficient for energy intake between two FFQs administered 3 months apart was 0.68, and for food groups (e.g., fruits, vegetables, and soft drinks) ranged from 0.62 to 0.79. Details of the FFQ have been described elsewhere (26). Factor analysis with principal components based on the FFQ was used to derive three dietary patterns (fruit and sweet foods dietary pattern, vegetable dietary pattern, and animal foods dietary pattern; Supplementary Table 1) (27). Anthropometric parameters, blood pressure, and blood tests are done by trained staff at the hospital. Body mass index (BMI) was calculated as weight/height^2^ (kg/m^2^). Metabolic syndrome (MetS) was defined according to the 2009 American Heart Association scientific criteria (28). Details of the various tests have been described in a previous study (29).

### Statistical analysis

All statistical analyses were performed using SAS software, version 9.4 (SAS Institute Inc., Cary, NC, United States). We summarized baseline characteristics based on *H. pylori* infection using the mean and 95% confidence interval (CI) for continuous variables and counts (with percentages) for categorical variables.

Propensity scores were calculated using a logistic regression model and the main covariates (refer to Table 1). We used nearest neighbor matching to match *H. pylori* infection and *H. pylori* uninfected patients in a 1:1 ratio with a caliper distance of 0.2 of the standard deviation of the logit of the PS (30). Standardized differences were used to assess the covariate balance after matching. An absolute standardized difference of less than 0.1 was considered negligible in the groups (31). After the cases and controls were randomly sorted, the control with the closest propensity score was selected for the first case. If the propensity score difference between the two was within the caliper range, the matching was successful; if it was outside the caliper range, the matching failed. If successful, the case and control were removed from the original database and matched to the next case. If no suitable matched control was found for the last case, it was deleted.

**Table 1:** Characteristics of the participants according to *Helicobacter pylori* infection status before and after matching in women.^a^

Characteristics	*H. pylori* infection status (before matching)		*P* ^b^	*H. pylori* infection status (after matching)		*P* ^b^
	No	Yes	No	Yes
No. of participants	1,426	1,287		1,025	1,025	
Age (y)	46.7 (46.2, 47.2)	48.2 (47.6, 48.7)	<0.0001	46.7 (46.2, 47.2)	46.5 (46.0, 47.1)	0.73
BMI (kg/m^2^)	23.6 (23.4, 23.7)	24.0 (23.8, 24.2)	<0.001	23.6 (23.4, 23.7)	23.7 (23.5, 23.8)	0.37
Total energy intake (kcal/day)	1875.9 (1848.5, 1903.7)	1835.4 (1807.6, 1863.6)	0.04	1876.6 (1849.4, 1904.1)	1868.6 (1841.5, 1896.0)	0.68
High-sensitivity C-reactive protein (ug/L)	1.32 (1.21, 1.42)	1.16 (1.05, 1.26)	0.03	1.31 (1.21, 1.42)	1.21 (1.10, 1.31)	0.14
PA (≥23MET × hour/week, %)	29.9	28.9	0.53	29.8	29.9	0.94
**Smoking status (%)**						
Current smoker	2.35	2.90	0.34	2.37	2.67	0.58
Ex-smoker	1.03	0.72	0.36	0.91	0.97	0.86
Non-smoker	96.6	96.4	0.71	96.7	96.4	0.57
**Alcohol drinking status (%)**						
Everyday drinker	0.74	0.98	0.45	0.75	0.80	0.85
Sometime drinker	43.1	44.8	0.34	43.15	41.13	0.23
Ex-drinker	8.12	8.10	0.98	8.17	9.30	0.24
Non-drinker	48.0	46.1	0.27	47.9	48.6	0.70
Educational level (≥college grade, %)	47.7	40.6	<0.0001	47.5	47.1	0.84
**Occupation (%)**						
Managers	32.6	27.0	<0.001	32.5	33.0	0.74
Professionals	11.7	11.1	0.59	11.7	12.0	0.76
Other	55.7	61.9	<0.001	55.9	55.0	0.61
Depressive symptoms score (≥45, %)	13.2	15.2	0.10	13.2	13.4	0.88
Metabolic syndrome	20.1	21.3	0.38	20.2	20.4	0.90
Amenorrhea (Yes, %)	31.2	34.3	0.05	31.2	29.9	0.40
Household income (≥10,000 Yuan, %)	41.6	38.2	0.04	41.7	41.5	0.92
**Family history of diseases (%)**						
CVD	35.8	36.9	0.51	35.9	35.3	0.70
Hypertension	56.6	54.6	0.24	56.6	57.0	0.79
Hyperlipidemia	0.17	0.06	0.35	0.00	0.00	-
Diabetes	27.9	27.4	0.74	27.8	27.8	1.00
“Sweets” dietary pattern score	−0.08 (−0.13, −0.04)	−0.07 (−0.11, −0.02)	0.64	−0.08 (−0.13, −0.04)	−0.08 (−0.12, −0.03)	0.82
“Vegetables” dietary pattern score	0.16 (0.12, 0.21)	0.13 (0.08, 0.17)	0.26	0.16 (0.12, 0.21)	0.16 (0.12, 0.21)	1.00
“Animal foods” dietary pattern score	−0.35 (−0.39, −0.32)	−0.33 (−0.36, −0.29)	0.32	−0.35 (−0.39, −0.31)	−0.34 (−0.38, −0.31)	0.77
Iodine in food (ug/d)	144.6 (139.6, 149.7)	146.1 (140.8, 151.3)	0.70	144.7 (139.6, 149.8)	148.0 (142.9, 153.1)	0.37

a Least square mean (95% confidence interval; all such values).

b Analysis of variance or Chi-square test.

Follow-up time was calculated from the date of measuring the baseline measurement of *H. pylori* infection to the date of the first diagnosis of subclinical hyperthyroidism, or the end of follow-up (31 December 2019), or loss to follow-up, whichever was earliest. Cox proportional hazards models were applied to calculate hazard ratios (HRs) and 95% CIs for the association between *H. pylori* infection and the risk of subclinical hyperthyroidism. The incidence of subclinical hyperthyroidism was used as the dependent variable. Three models were developed. In Model 1, we adjusted for age, BMI, smoking status, drinking status, education levels, employment status, household income, depressive symptoms score, PA, family history of diseases (including CVD, hypertension, hyperlipidemia, and diabetes), total energy intake, high-sensitivity C-reactive protein (hsCRP), MetS, and amenorrhea status. The total iodine intake in food accounts for a high proportion of iodine intake, as high as 42.7% in the Tianjin population (21), thus, in Model 2, we further adjusted the total iodine intake in food. A study (32) has shown that diet in daily life will have an impact on *H. pylori* infection, thus, we have adjusted dietary patterns in a final model.

Potential interactions between *H. pylori* infection and main covariates were assessed by adding cross-product terms to the multivariable Cox models. We stratified the participants by potential effect modifiers including age, BMI, physical activity, amenorrhea status, education level, household income, and Mets status. To examine the robustness of our results, we conducted two sensitivity analyses: (1) we substituted adjustment for waist circumference (WC) was measured with BMI to investigate whether abdominal adiposity, compared with overall adiposity, changed the observed associations. (2) We included participants who had a history of cancer or CVD. All tests were two-tailed and *p* < 0.05 was defined as statistically significant.

## Results

**Figure 2:**
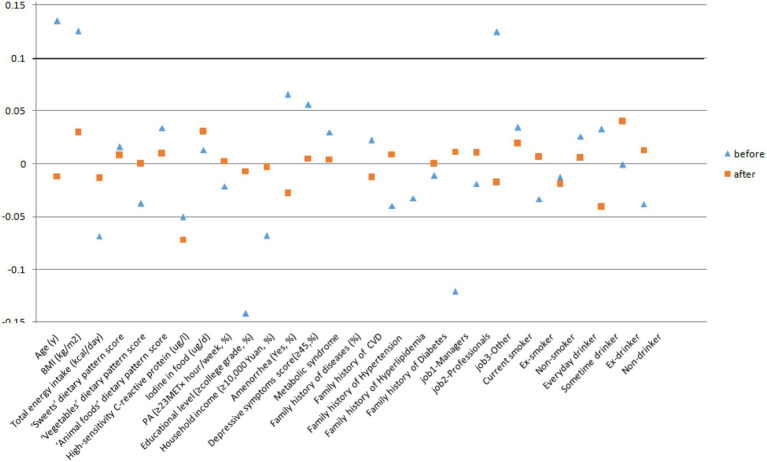
Standardized mean differences of covariances before and after propensity score matching.

Characteristics of participants according to *H. pylori* infection status before and after PSM are shown in Table 1 and Figure 2. Among 2,713 participants who were available to be analyzed before PSM, 47.4% were classified as infecting *H. pylori.* Participants with *H. pylori* infection status tended to be older, and have higher BMI. Moreover, they had lower total energy intake, hsCRP, educational level, and monthly household income, and they were more likely to be managers and had amenorrhea status. After PSM, there were 1,025 PS-matched pairs of *H. pylori* infection women were generated and showed no significant baseline differences in any characteristic. Between May 2013 and December 2019, a total of 2,050 participants completed the follow-up analysis for subclinical hyperthyroidism. In the total population, 55 first incident cases of subclinical hyperthyroidism occurred, the incidence rate of subclinical hyperthyroidism was 7.56 per 1,000 person-years across the 6-year follow-up period (range: 2–6 years; median: 3.55 years).

Table 2 presents the associations between *H. pylori* infection and the risk of subclinical hyperthyroidism. In the analysis of *H. pylori* infection status before PSM, after adjustment for age, BMI, sociodemographic and lifestyle factors as well as for Mets status (Model 1), HR (95% CI) for subclinical hyperthyroidism was 2.07 (1.06, 4.03; *p* = 0.033). Further adjustments were made for total iodine intake in food, HRs (95% CIs) for subclinical hyperthyroidism were 2.05 (1.05, 4.02; *p* = 0.036), and we observed similar results. In the fully adjusted model further adjusted for the dietary quality (three major dietary patterns), these associations were not altered, and HR (95% CI) for subclinical hyperthyroidism was 2.06 (1.05, 4.03; *p* = 0.035). After *H. pylori* infection status PSM, we observed more obvious associations between *H. pylori* infection and the risk of subclinical hyperthyroidism. In Model 1, HR (95% CI) for subclinical hyperthyroidism was 2.34 (1.28, 4.27; *p* = 0.006). In Model 2, HR (95% CI) for subclinical hyperthyroidism was 2.41 (1.32, 4.40; *p* = 0.004). In the fully adjusted model further adjusted for the dietary quality (three major dietary patterns), these associations were not altered, HR (95% CI) for subclinical hyperthyroidism was 2.49 (1.36, 4.56; *p* = 0.003).

**Table 2:** Association between *Helicobacter pylori* infection status and subclinical hyperthyroidism before and after matching in women.

	*H. pylori* infection status (before matching)		*P* ^a^	*H. pylori* infection status (after matching)		*P* ^a^
	No	Yes		No	Yes	
No. of subjects	1,426	1,287		1,025	1,025	
Number of cases	25	37		19	36	
Person-years	4,124	3,828		3,640	3,635	
Model 1^b^	1.00 (reference)	2.07 (1.06, 4.03)^c^	0.033	1.00(reference)	2.34 (1.28, 4.27)	0.006
Model 2^d^	1.00(reference)	2.05 (1.05, 4.02)	0.036	1.00(reference)	2.41 (1.32, 4.40)	0.004
Model 3^e^	1.00(reference)	2.06 (1.05, 4.03)	0.035	1.00(reference)	2.49 (1.36, 4.56)	0.003

a Analysis by Cox proportional hazards regression model.

b Model 1 was adjusted for age (years, continuous), BMI (kg/m^2^, continuous), smoking status (current, former, or never), drinking status (everyday drinker, sometimes drinker, ex-drinker, or non-drinker), education levels (categorical: < or ≥college graduate), employment status (categorical: managers, professionals and other), household income (categorical: ≤ or >10,000 Yuan), depressive symptoms score (categorical: < or ≥45), physical activity [(categorical: < or ≥23) metabolic equivalent-h/week], family history of diseases [including cardiovascular disease, hypertension, hyperlipidemia, and diabetes (each yes or no)], total energy intake (continuous: kcal/d), high-sensitivity C-reactive protein (mg/L), metabolic syndrome (categorical: yes or no), amenorrhea status (categorical: yes or no).

c Hazard ratios (95% confidence interval; all such values).

d Model 2 was further adjusted for variable total iodine intake in food(ug/d).

e Model 3 was adjusted for variables in Model 3 plus “fruits and sweet” dietary pattern score, “healthy” dietary pattern score, and “animal foods” dietary pattern score.

The analyses were stratified by age (<40 or ≥40 years), BMI (< 24.0 or ≥24.0), physical activity (<23 or ≥23 MET-h/wk), MetS (yes or no), amenorrhea status (yes or no), an education level (college graduate or not), and household income (<10,000 or ≥10,000 yuan). The associations between *H. pylori* infection status and the risk of subclinical hyperthyroidism were generally similar across all subgroups (Table 3). All interactions were not statistically significant (*P* for interaction > 0.05), except for age (*P* for interaction = 0.05). Stratified analyses indicated that *H. pylori* infection was associated with subclinical hyperthyroidism in individuals aged ≥ 40 but not in individuals aged < 40. Similar results were also found in the sensitivity analysis (Table 4). When we adjusted for WC instead of BMI, the full model-adjusted HRs 95% CI for subclinical hyperthyroidism in women were 2.48 (1.36, 4.54; *p* = 0.003), and the associations did not substantially change. When we included participants with a history of cancer or CVD, the analyses yielded very similar results, the adjusted HRs (95% CIs) of subclinical hyperthyroidism were 2.03 (1.13, 3.64; *p* = 0.019).

**Table 3:** Association between *Helicobacter pylori* infection status and risk of Subclinical hyperthyroidism in women stratified by major covariates.

	*H. pylori* infection status		*P* ^a^	*P* for interaction^b^
	No	Yes		
Age (years)				0.048
≥40	1.00 (reference)	2.85 (1.45, 5.61)^c^	0.002	
<40	1.00 (reference)	0.70 (0.08, 6.00)	0.747	
BMI (kg/m^2^)				0.453
≥24.0	1.00 (reference)	2.73 (1.03, 7.21)	0.042	
<24.0	1.00 (reference)	2.94 (1.28, 6.78)	0.011	
PA (MET × hour/week)				0.140
≥23.0	1.00 (reference)	2.94 (0.97, 8.93)	0.057	
<23.0	1.00 (reference)	2.29 (1.08, 4.87)	0.032	
Amenorrhea status				0.739
Yes	1.00 (reference)	2.03 (0.62, 6.69)	0.244	
No	1.00 (reference)	2.57 (1.21, 5.42)	0.014	
Education level				0.387
≥College	1.00 (reference)	2.02 (0.85, 4.81)	0.111	
<College	1.00 (reference)	3.13 (1.23, 7.99)	0.017	
Household income, yuan				0.618
≥10,000	1.00 (reference)	2.84 (1.16, 6.92)	0.022	
<10,000	1.00 (reference)	2.26 (0.93, 5.47)	0.071	
Metabolic syndrome				0.981
Yes	1.00 (reference)	1.99 (0.28, 14.1)	0.490	
No	1.00 (reference)	2.56 (1.29, 5.06)	0.007	

a Multivariable Cox proportional regression was adjusted for age (years, continuous), BMI (kg/m^2^, continuous), smoking status (current, former, or never), drinking status (everyday drinker, sometime drinker, ex-drinker, or non-drinker), education levels (categorical: < or ≥college graduate), employment status (categorical: managers, professionals and other), household income (categorical: ≤ or >10,000 Yuan), depressive symptoms score (categorical: < or ≥45), physical activity [(categorical: < or ≥23) metabolic equivalent-h/week], family history of diseases (including cardiovascular disease, hypertension, hyperlipidemia, and diabetes; categorical: each yes or no), total energy intake (continuous: kcal/d), high-sensitivity C-reactive protein (mg/L), metabolic syndrome (categorical: yes or no), amenorrhea status(categorical: yes or no), total iodine intake in food(ug/d), three dietary patterns (“sweet” dietary pattern score, “vegetable” dietary pattern score, “animal food” dietary pattern score).

b *P* for interaction was calculated using the likelihood ratio test.

c HRs (95% CIs; all such values).

**Table 4:** Sensitivity analysis of the association between *Helicobacter pylori* infection status and Subclinical hyperthyroidism in women.

	*H. pylori* infection status		*P* ^a^
	No	Yes	
**Sensitivity analysis 1** ^b^			
No. of subjects	1,025	1,025	
Number of cases	19	36	
Person-years	3,640	3,635	
Model 3^c^	1.00(reference)	2.48 (1.36, 4.54)^d^	0.003
**Sensitivity analysis 2** ^**e**^			
No. of subjects	1,084	1,084	
Number of cases	21	34	
Person-years	3,846	3,782	
Model 3^c^	1.00(reference)	2.03 (1.13, 3.64)	0.019

a Analysis by Cox proportional hazards regression model.

b Adjusted for waist circumference instead of body mass index.

c Model 3 was adjusted for age (years, continuous), BMI (kg/m^2^, continuous), smoking. status (current, former, or never), drinking status (everyday drinker, sometimes drinker, ex-drinker, or non-drinker), education levels (categorical: < or ≥ college graduate), employment status (categorical: managers, professionals, and other), household income (categorical: ≤ or > 10,000 Yuan), depressive symptoms score (categorical: < or ≥45), physical activity [(categorical: < or ≥23) metabolic equivalent-h/week], family history of diseases [including cardiovascular disease, hypertension, hyperlipidemia, and diabetes (each yes or no)], total energy intake (continuous: kcal/d), high-sensitivity C-reactive protein (mg/L), metabolic syndrome (categorical: yes or no), amenorrhea status(categorical: yes or no), total iodine intake in food(ug/d), three dietary patterns (“sweet” dietary pattern score, “vegetable” dietary pattern score, “animal food” dietary pattern score).

d Hazard ratios (95% confidence interval; all such values).

e Included participants with a history of cancer or CVD.

## Discussion

In this large-scale prospective study of a Chinese adult population, we have assessed the association between *H. pylori* infection and the incidence of subclinical hyperthyroidism in women. Considering the potential effect of iodine on subclinical hyperthyroidism, we conducted this cohort study in Tianjin, an iodine-replete area, and further adjusted for total iodine intake in food in Model 2. Meanwhile, dietary factors are not only associated with *H. pylori* infection (33) but also affect the risk of subclinical hyperthyroidism (34), however, no previous cohort study adjusted for dietary intake when exploring the associations between *H. pylori* infection and the incidence of subclinical hyperthyroidism, indicating that the risk effect of *H. pylori* infection on incident subclinical hyperthyroidism might be misestimated. In this large prospective cohort study, we adjusted for dietary factors in the form of three dietary patterns in Model 3, thus, can prevent the influence of dietary factors on the association between *H. pylori* infection and subclinical hyperthyroidism. We found that *H. pylori* infection was associated with an increased risk of incidents of subclinical hyperthyroidism independent of dietary factors in women, a series of sensitivity and subgroup analyses supported these results. To the best of our knowledge, this study is the first prospective cohort investigation regarding the association between *H. pylori* infection and subclinical hyperthyroidism independent of dietary factors in women.

The present results suggested that *H. pylori* infection was significantly associated with subclinical hyperthyroidism in women, which seems to be in agreement with previous studies (16, 35). Although the exact mechanisms of *H. pylori* infection in subclinical hyperthyroidism have not been elucidated, some evidence may explain the association. First, it was recently discovered that *H. pylori* strains can express fucosylated Lewis determinants, which are widely shared by different host tissues and may stimulate an autoimmune response that could potentially damage the thyroid gland (36). Second, a previous study has shown that a homologous 11-residue peptide in both gastric parietal cell antigen and thyroid peroxidase suggests the existence of an epitope common to both antigens (37). Therefore, antibodies are produced during *H. pylori* infection may cross-react with thyroid antigens, resulting in subclinical hyperthyroidism (38). Furthermore, evidence that *H. pylori* infection can increase the risk of subclinical hyperthyroidism also comes from the strong correlation between IgG anti-*H. pylori* antibodies and thyroid autoantibodies, as well as the observation that thyroid auto-antibodies levels gradually decrease after eradication of *H. pylori* infection (39). Taken together, these studies provide potential explanations linking *H. pylori* infection and subclinical hyperthyroidism.

We found a significant association between *H. pylori* infection and subclinical hyperthyroidism in middle-aged and elderly women but not in younger women. This association may be caused by two reasons. First, the successful colony of *H. pylori* in the stomach requires age-related gastric physiology and special characteristics related to the host (40), and *H. pylori*-related diseases are increasing with increasing age (41). Second, the thyroid gland would undergo important functional changes during aging, the clinical course of thyroid diseases is different between the elderly and the young. A previous study showed a degree of insensitivity in thyroid cells in the anterior pituitary gland in older adults, occurring with age, mainly manifested as the decrease of TSH secretion of thyrotropin-releasing hormone (TRH) in the elderly, and the serum TSH level is usually higher in older than in young people in response to the decrease of thyroid hormone concentration (42). Further exploration is required to clarify this issue.

The major strengths of this study are it first assessed the association between *H. pylori* infection and subclinical hyperthyroidism in a large-scale adult population from an iodine-replete area. It is a prospective dynamic study design, which allowed us to examine the long-term effect of *H. pylori* infection on thyroid function before the occurrence of subclinical hyperthyroidism. Furthermore, we applied the PSM in the *H. pylori*-infected and -uninfected so we could observe the balance of confounding factors between groups more intuitively in comparing matched groups. Thus, the distribution of covariates between the two groups was balanced and the bias in the observational study was reduced. Moreover, we performed several sensitivity analyses to confirm the robustness of the findings. Nonetheless, several biases and limitations should be considered in the present study. First, although our analyses adjusted for a considerable number of confounders, we could not rule out residual confounding by other unmeasured or unknown factors. Second, the clinical outcome of *H. pylori* infection may be determined by a combination of virulence among *H. pylori* strains, duration of infection, host genetic polymorphisms, specific host–microbe interactions, and environmental factors (43). However, our study was based on data from health check-ups; *H. pylori* strains, eradication of *H. pylori* infection, and genome-wide association studies (GWAS) were not investigated. CagA-positive strains are endowed with an enhanced inflammatory potential (44) and affect patients with hyperthyroid Graves’s disease more frequently (45). Previous studies supported the finding that eradicating *H. pylori* infection could lead to the restitution of the cellular immune response (46) and reduce thyroid autoantibodies (14). In addition, one study found the presence of a gene encoding an endogenous peroxidase in the dissected chromosome of an *H. pylori* strain (47). Therefore, the lack of data on *H. pylori* might modify the strength of the study results, underestimating the observed associations. Third, although subjects with a self-reported history of all types of thyroid disease, including positive thyroid autoantibodies, were excluded based on a detailed structured questionnaire, serum thyroid autoantibodies were not measured in the current study. Previous reviews (48) have shown that positive thyroid autoantibodies can predict the risk of future thyroid dysfunction, and studies have shown that thyroid autoantibodies reduce the risk of *H. pylori* infection after eradication (39), suggesting that the lack of data on thyroid autoantibodies may lead to underestimation of the risk of *H. pylori* infection to subclinical hyperthyroidism. Finally, *H. pylori* infection rates vary according to geographic region (49), and our study was performed in the general adult Chinese population, therefore, it is possible that our results cannot be generalized to other populations. Further studies are needed to verify the results in other populations.

## Conclusion

In conclusion, the findings of this population-based cohort study suggested that *H. pylori* infection was associated with the risk of subclinical hyperthyroidism independent of dietary factors in adult women. More studies should be performed on different populations to confirm these findings.

## Data availability statement

The original contributions presented in the study are included in the article/Supplementary material, further inquiries can be directed to the corresponding author/s.

## Ethics statement

The studies involving human participants were reviewed and approved by UMIN Clinical Trials Registry. The patients/participants provided their written informed consent to participate in this study.

## Author contributions

JZ and XH analyzed data and wrote the paper. SW, FZ, YG, GM, QZ, LL, HW, SZ, TZ, XW, SS, MZ, QJ, and KS conducted research. KN designed research and had primary responsibility for final content. All authors read and approved the final manuscript.

## Funding

This study was supported by grants from the National Natural Science Foundation of China (No. 81941024, 81872611, 82103837, and 81903315), Tianjin Major Public Health Science and Technology Project (No. 21ZXGWSY00090), National Health Commission of China (No. SPSYYC 2020015), Food Science and Technology Foundation of Chinese Institute of Food Science and Technology (No. 2019-12), 2014 and 2016 Chinese Nutrition Society (CNS) Nutrition Research Foundation—DSM Research Fund (Nos. 2016-046, 2014-071 and 2016-023), China.

## Conflict of interest

The authors declare that the research was conducted in the absence of any commercial or financial relationships that could be construed as a potential conflict of interest.

## Publisher’s note

All claims expressed in this article are solely those of the authors and do not necessarily represent those of their affiliated organizations, or those of the publisher, the editors and the reviewers. Any product that may be evaluated in this article, or claim that may be made by its manufacturer, is not guaranteed or endorsed by the publisher.

## Abbreviations

*H. pylori*: *Helicobacter pylori*
Subclinical hyperthyroidism: Subclinical thyroid dysfunction
FT3: Free triiodothyronine
FT4: Free thyroxine
HR: Hazard ratio
THs: Thyroid hormones
T3: Triiodothyronine
T4: Thyroxine
TSH: Thyroid-stimulating hormone
CI: Confidence interval
CVD: Cardiovascular disease
AITDs: Autoimmune thyroid diseases
TCLSIH: Tianjin Chronic Low-grade Systemic Inflammation and Health
^13^C-UBT: ^13^C urea breath test
BMI: Body mass index
PA: Physical activity
FFQ: Food Frequency Questionnaire
WC: Waist circumference
FBG: Fasting blood glucose
TG: Triglycerides
TC: Total cholesterol
LDL: Low-density lipoprotein cholesterol
HDL: High-density lipoprotein cholesterol
BP: Blood pressure
