# Supplementary material for: Helicobacter pylori infection increase the risk of subclinical hyperthyroidism in middle-aged and elderly women independent of dietary factors: Results from the Tianjin chronic low-grade systemic inflammation and health cohort study in China
Source: Front Nutr. 2023 Mar 6;10:1002359. doi: 10.3389/fnut.2023.1002359 (PMC10025335; doi:10.3389/fnut.2023.1002359)
Supplement: Supplementary file 1 [file Table_1.DOCX]

| **Supplementary Table 1. The factor loadings of primary food items of dietary patterns ^a^** | | | | | |
| --- | --- | --- | --- | --- | --- |
| Fruit and sweet foods pattern | | Vegetable foods pattern | | Animal foods pattern | |
| Food items | Factor loadings | Food items | Factor loadings | Food items | Factor loadings |
| Strawberry, kiwi fruit, persimmon | 0.66 | Chinese cabbage | 0.64 | Animal offal (except for animal liver) | 0.71 |
| Grape | 0.63 | Cucumber | 0.64 | Animal liver | 0.66 |
| Pineapple | 0.63 | Green vegetable | 0.63 | Animal blood | 0.65 |
| Western-style pastry, cakes | 0.57 | Celery | 0.61 | Preserved egg | 0.64 |
| Peach | 0.57 | Pumpkin, carrot | 0.55 | Instant noodle | 0.60 |
| Sweets, candied fruits | 0.56 | Tomato (including the ketchup) | 0.55 | Sausage | 0.58 |
| Pear | 0.56 | Eggplant | 0.55 | Pork skin | 0.57 |
| Chinese cakes | 0.55 | Chinese watermelon | 0.53 | Wonton | 0.55 |
| Ice cream | 0.52 | Egg | 0.52 | Sea fish | 0.54 |
| Watermelon | 0.51 | Raw vegetables (except for | 0.52 | Freshwater fish | 0.54 |
| Banana | 0.51 | Mushroom | 0.51 | Seafood (shellfish, squid, shrimp) | 0.52 |
| Cookies | 0.51 | Bell peppers | 0.50 | Carbonated beverage | 0.49 |
| Sea-plant | 0.50 | Soya bean products | 0.49 | Miscellaneous sauce noodles | 0.47 |
| Salted eggs | 0.50 | Coarse cereals | 0.49 | Sweets, candied fruits | 0.42 |
| Lotus root | 0.49 | Potato (except for sweet potato) | 0.49 | Ice cream | 0.42 |
| ^a^ Only shows the top 15 foods in the Factor loadings | | | | | |
